# Veno-venous extracorporeal CO_2_ removal for the treatment of severe respiratory acidosis: pathophysiological and technical considerations

**DOI:** 10.1186/cc13928

**Published:** 2014-06-17

**Authors:** Christian Karagiannidis, Kristin Aufm Kampe, Fernando Suarez Sipmann, Anders Larsson, Goran Hedenstierna, Wolfram Windisch, Thomas Mueller

**Affiliations:** 1 Department of Pneumology and Critical Care Medicine, Cologne-Merheim Hospital, Kliniken der Stadt Köln gGmbH, Witten/Herdecke University Hospital, Ostmerheimer Strasse 200, Cologne D-51109, Germany; 2 Hedenstierna Laboratory, Anesthesiology and Intensive Care, Department of Surgical Sciences, Uppsala University, Uppsala, Sweden; 3 CIBER de Enfermedades Respiratorias, Instituto de Salud Carlos III, Madrid, Spain; 4 Department of Pneumology, University Hospital Freiburg, Killianstr.5, Freiburg D-79106, Germany; 5 Department of Internal Medicine II, University Hospital of Regensburg, Franz-Josef-Strauss-Allee 11, Regensburg 93053, Germany

## Abstract

**Introduction:**

While non-invasive ventilation aimed at avoiding intubation has become the modality of choice to treat mild to moderate acute respiratory acidosis, many severely acidotic patients (pH <7.20) still need intubation. Extracorporeal veno-venous CO_2_ removal (ECCO_2_R) could prove to be an alternative. The present animal study tested in a systematic fashion technical requirements for successful ECCO_2_R in terms of cannula size, blood and sweep gas flow.

**Methods:**

ECCO_2_R with a 0.98 m^2^ surface oxygenator was performed in six acidotic (pH <7.20) pigs using either a 14.5 French (Fr) or a 19Fr catheter, with sweep gas flow rates of 8 and 16 L/minute, respectively. During each experiment the blood flow was incrementally increased to a maximum of 400 mL/minute (14.5Fr catheter) and 1000 mL/minute (19Fr catheter).

**Results:**

Amelioration of severe respiratory acidosis was only feasible when blood flow rates of 750 to 1000 mL/minute (19Fr catheter) were used. Maximal CO_2_-elimination was 146.1 ± 22.6 mL/minute, while pH increased from 7.13 ± 0.08 to 7.41 ± 0.07 (blood flow of 1000 mL/minute; sweep gas flow 16 L/minute). Accordingly, a sweep gas flow of 8 L/minute resulted in a maximal CO_2_-elimination rate of 138.0 ± 16.9 mL/minute. The 14.5Fr catheter allowed a maximum CO_2_ elimination rate of 77.9 mL/minute, which did not result in the normalization of pH.

**Conclusions:**

Veno-venous ECCO_2_R may serve as a treatment option for severe respiratory acidosis. In this porcine model, ECCO_2_R was most effective when using blood flow rates ranging between 750 and 1000 mL/minute, while an increase in sweep gas flow from 8 to 16 L/minute had less impact on ECCO_2_R in this setting.

## Introduction

Non-invasive ventilation (NIV) has become the modality of choice to treat mild to moderate respiratory acidosis (pH ≥7.20) due to chronic obstructive pulmonary disease (COPD) exacerbation, since it has been shown to avoid intubation and intubation-related complications, resulting in reduced ICU-mortality [1]. However, real-life observations have revealed high rates of both intubation and mortality in patients with COPD exacerbation and severe acute hypercapnic respiratory failure [2-4]. This is related to severe respiratory acidosis, well-established contraindications of NIV, lack of staff training and the presence of co-morbidities hindering the successful application of NIV [5,6].

This has led to the attempt of extracorporeal carbon dioxide (CO_2_) removal (ECCO_2_R) in patients presenting with acute hypercapnic respiratory failure. ECCO_2_R systems have been successfully used to reduce invasiveness of mechanical ventilation and, therefore, ventilator-induced lung injury in acute respiratory distress syndrome (ARDS) patients [7-12]. Recently, ECCO_2_R by means of a pumpless, arterio-venous extracorporeal lung-assist has been shown to preclude the need for intubation and invasive mechanical ventilation in a case–control study in COPD patients with acute-on-chronic respiratory failure [13]. In addition, previous research has demonstrated that this technique is capable of eliminating approximately 50% of the calculated CO_2_ production, with rapid normalization of respiratory acidosis [14]. Importantly, these systems are driven by the arterio-venous pressure gradient; thus, cannulation of arterial vessels (most commonly the femoral artery), coupled with cannulation of the corresponding vein on the contralateral limb, is necessary for driving the system [13-15]. Based on these preconditions, there are clearly defined contraindications and several complications directly related to arterial cannulation, such as bleeding, hematoma or aneurysm at the insertion site, and ischemia and/or compartment syndrome of the lower limb [15,16].

Interestingly, a recent study reported the successful application of a pump-driven veno-venous system using a 15.5 French (Fr) dual-lumen catheter with a mean blood flow of 431 ml/minute [17]. Even though this was a pilot study testing the feasibility of this new approach, the study showed the potential of these veno-venous systems to improve respiratory acidosis without requiring arterial cannulation. However, patients only had moderate respiratory acidosis, and it remains unclear how these results translate to more severe respiratory acidosis. Accordingly, clinical experience suggests that substantially higher flow rates are needed to correct severe respiratory acidosis (pH <7.2) [18]. In this regard, the physiological relationships between cannula size, blood flow, sweep gas flow and gas transfer capacity, respectively, are still largely unknown. For this reason, more physiological data on these issues are needed before the promising technique of miniaturized veno-venous ECCO_2_R can be tested in a broader clinical setting. Therefore, we set up an animal study that aimed to elucidate the relationships between cannula size, blood flow and sweep gas flow, respectively, in pigs with experimentally-induced severe respiratory acidosis that mimicked severe acute hypercapnic respiratory failure with pH values between 7.0 and 7.2.

## Material and methods

### Extracorporeal CO_2_ removal techniques

For ECCO_2_R a pump assisted lung protection (PALP) System® (Maquet Cardiopulmonary Care, Rastatt, Germany) based on the Cardiohelp® platform was used. The oxygenator has an area of 0.98 m^2^ with a poly methylpentene membrane lacking heat exchange fibers. The priming volume of the whole system is 247 ml. The PALP system® was primed with normal saline solution. Heparin (1,000 IE) was added to the running system.

For venous access, a 14.5Fr hemodialysis catheter (Fresenius Medical Care, Bad Homburg Germany) was used during Experiments 1 and 2, while a 19Fr Bicaval Avalon ELITE Dual Lumen Cannula® (Maquet Cardiopulmonary Care, Rastatt, Germany) was implanted for Experiments 3, 4 and 5. Pigs were anticoagulated with heparin during extracorporeal treatment. For all experiments, the sweep gas flow was applied with 100% oxygen by a Flow-i Anesthesia Delivery System (Maquet Critical Care, Solna, Sweden); in a subset of animals the sweep gas flow was provided with room air.

### Animal model

The study was approved by the Animal Research Committee of Uppsala University in Sweden (ethical approval number: C265/12). Pigs (body weight = 39.8 ± 2.9 kg) were anesthetized with IV ketamine 25 to 50 mg/kg/hour, midazolam 90 to 180 μg/kg/hour, fentanyl 3 to 6 μg/kg/hour and rocuronium 2.5 to 5.0 mg/kg/hour. The trachea was intubated with a cuffed endotracheal tube (inner diameter, 7 mm). The pigs were ventilated with a Servo-i ventilator (Maquet Critical Care, Solna, Sweden). Body temperature was kept at 37°C throughout the whole study period by the use of a warm blanket. Arterial blood was taken from the left carotid artery. The estimated CO_2_ production is about 7 ml/kg/minute in pigs [19], that is, a CO_2_ production of approximately 280 ml/minute, which is comparable to an adult human.

### Study design

ECCO_2_R was performed in six pigs following intubation, mechanical ventilation and induction of respiratory acidosis by increased dead space ventilation. In detail, anatomical dead space was increased by adding a further tube between the endotracheal tube and the ‘Y’ piece of the ventilator circuit. The length of the additional tube was titrated until respiratory acidosis was induced with a pH value of 7.0 to 7.1. Pigs were ventilated in a volume controlled mode with a tidal volume of 360 ml, a positive end expiratory pressure (PEEP) of 6 cm H_2_O and a breathing frequency of 15/minute. Dead space fractions and CO_2_ elimination were measured with a NICO monitor (Philips, Wallingford, CT, USA), where airway flow and CO_2_ signals were monitored by mainstream sensors placed between the endotracheal tube and the ‘Y’ piece of the ventilator circuit. Dead space was computed as PCO_2_et–PeCO_2_/PCO_2_et, where PCO_2_et represents the end-tidal partial pressure of CO_2_ and PeCO_2_ the mixed expired CO_2_. Dead space fraction was measured in the first pig by the Bohr equation, with a resulting fraction of 0.85. This dead space fraction was subsequently maintained for the entire duration of the experimental period.

Five experiments were performed in each pig in a standardized fashion. First, ECCO_2_R was performed via a 14.5Fr hemodialysis catheter inserted into the right jugular vein (experiments 1 and 2). Subsequently, ECCO_2_R was performed via the 19Fr catheter following re-catheterization of the same right jugular vein (experiments 3, 4, and 5). Correct placement of the 19Fr cannula was confirmed by echocardiography. Equal conditions were used across all experiments (lasting at least 60 minutes), with each experiment starting at the pre-determined acidotic conditions.

During experiments 1 to 4, blood flow rates were increased in a stepwise fashion, while sweep gas flow was maintained (Table 1). Each step lasted 15 minutes in order to achieve equilibrium conditions, with all measurements taken at the end of this 15-minute period.

**Table 1:** Experimental set-up (experiments 1 to 5)

**Parameter**	**Experiment**	**Experiment**	**Experiment**	**Experiment**	**Experiment**
	**1**	**2**	**3**	**4**	**5**
**Catheter size** (French)	14.5	14.5	19	19	19
**Sweep gas flow** (L/min)	8	16	8	16	2 to 10
**Blood flow** (ml/min)	0	0	0	0	1,000
	200	200	250	250	
	400	400	500	500	
			750	750	
			1,000	1,000	

During experiment 5, sweep gas flow was changed by increasing flow in a stepwise approach (Table 1), while blood flow was maintained at a rate of 1,000 ml/min. Each step lasted 15 minutes in order to achieve equilibrium conditions.

### CO_2_ and blood gas measurement

CO_2_ was measured in the mainstream of the exhaust/sweep-gas outlet of the oxygenator by the Vaisala Carbocap GM 70 (Vaisala, Bonn, Germany). A water trap was integrated into the circuit before CO_2_ measurement. CO_2_ removal was calculated by multiplying the sweep gas flow with CO_2_ in Vol% in the exhaust/sweep-gas outlet. CO_2_ was measured with 0.04 Vol% in fresh air. Blood gas analysis was performed with an ABL 800, Radiometer, (Copenhagen, Denmark).

### Statistics

For statistical analysis, GraphPad Prism 5 for Macintosh computer (La Jolla, CA USA) was used. Data were tested for normality using the Kolmogorov-Smirnov test with a cut-off *P* value of <0.05. Normally-distributed data are expressed as mean and standard deviation.

## Results

**Figure 1:**
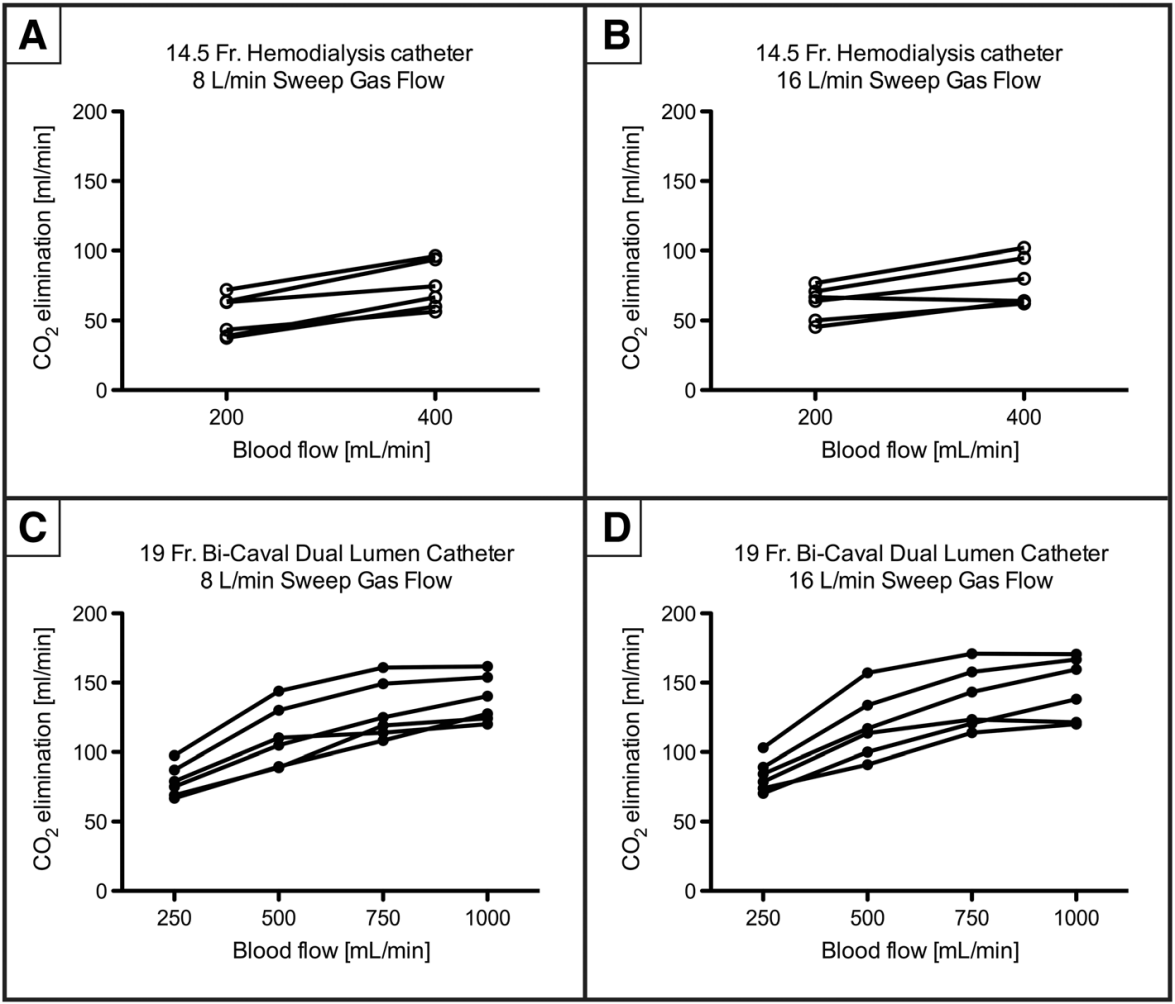
**Elimination of carbon dioxide (CO**_**2**_**) depending on blood flow. A)** 14.5Fr catheter; 8 L/minute sweep gas flow. **B)** 14.5Fr catheter; 16 L/minute sweep gas flow. **C)** 19Fr catheter; 8 L/minute sweep gas flow. **D)** 19Fr catheter; 16 L/minute sweep gas flow. Fr, French.

**Figure 2:**
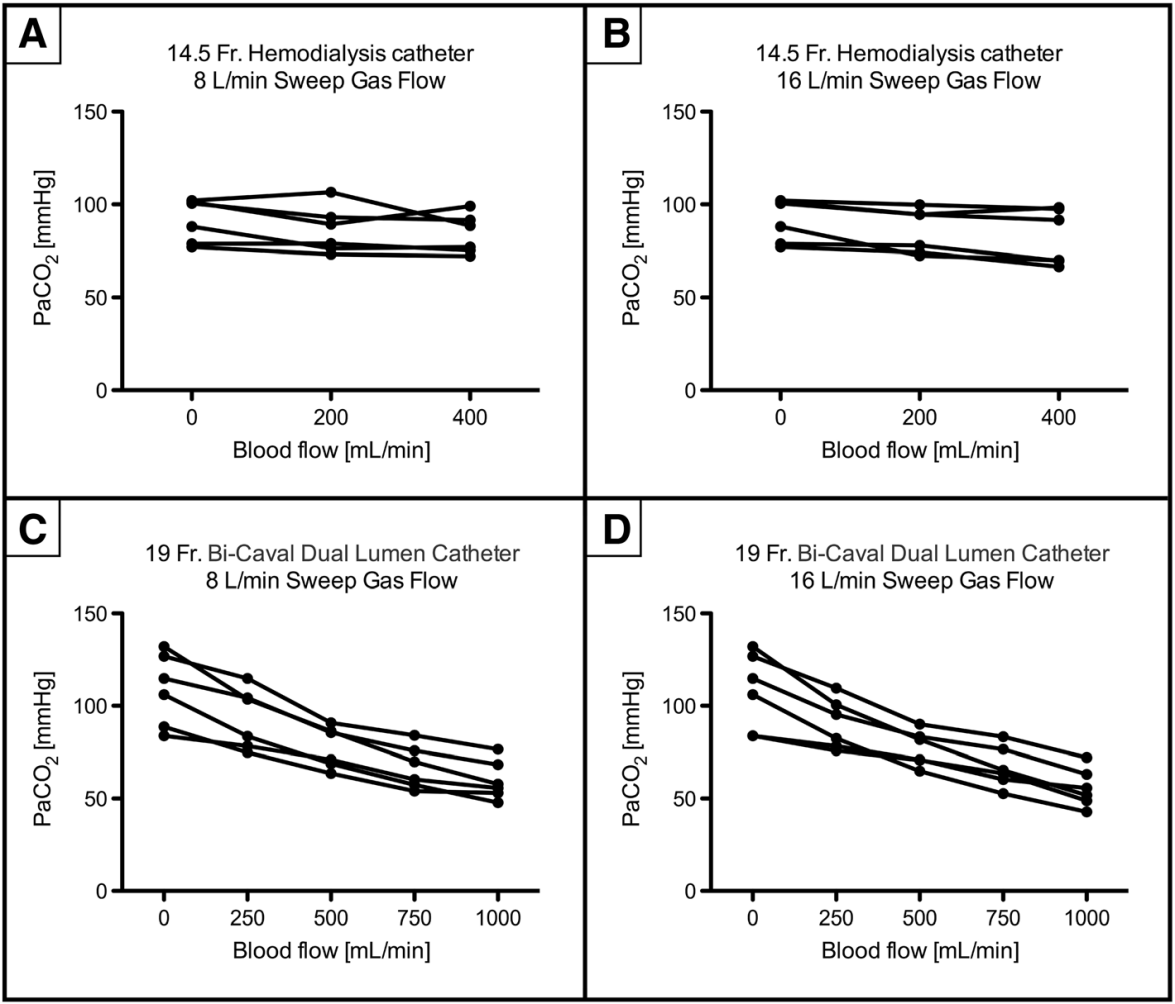
**Partial pressure of arterial carbon dioxide (PaCO**_**2**_**) depending on blood flow. A)** 14.5Fr catheter; 8 L/minute sweep gas flow. **B)** 14.5Fr catheter; 16 L/minute sweep gas flow. **C)** 19Fr catheter; 8 L/minute sweep gas flow. **D)** 19Fr catheter; 16 L/minute sweep gas flow. Fr, French.

**Figure 3:**
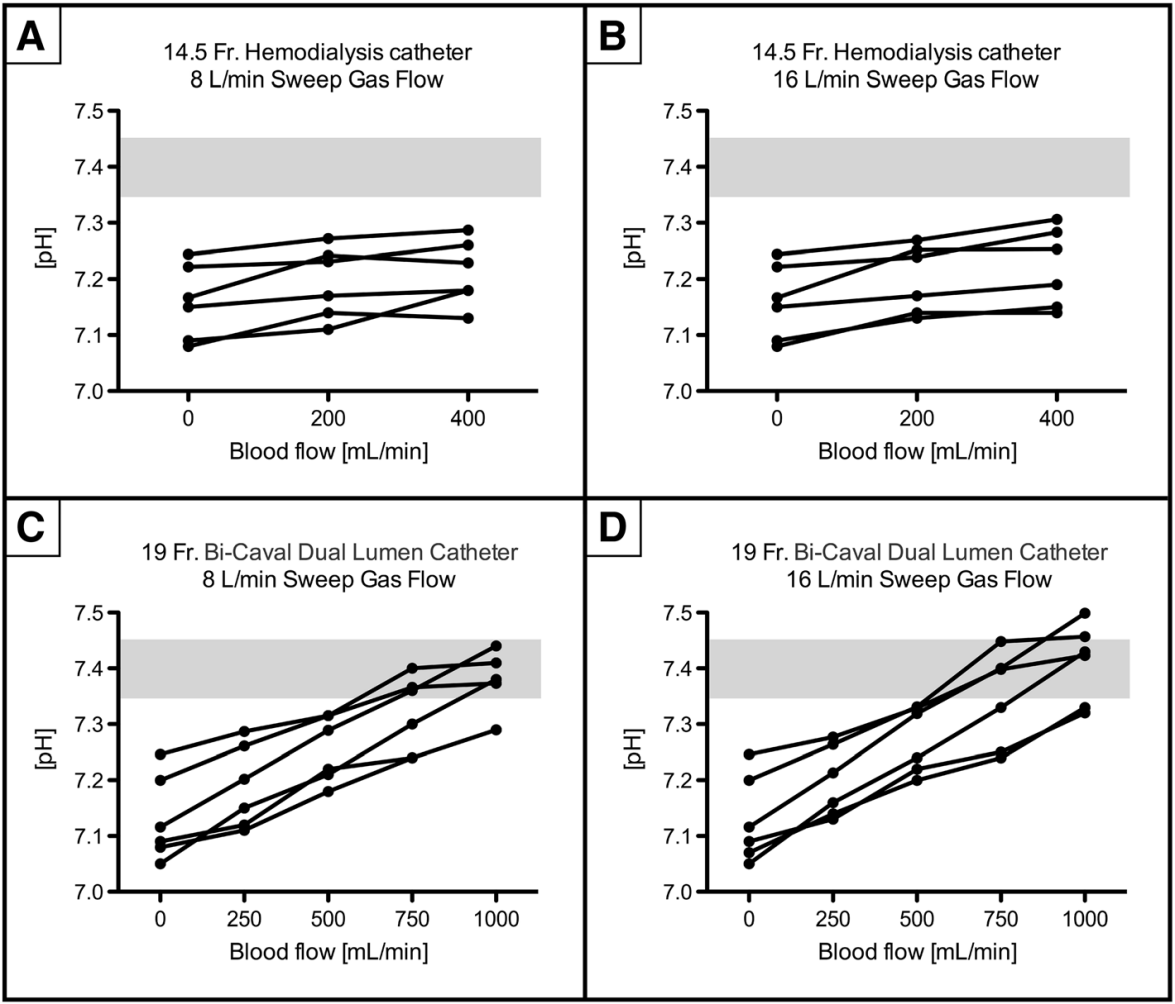
**pH dependence on blood flow. A)** 14.5Fr catheter; 8 L/minute sweep gas flow. **B)** 14.5Fr catheter; 16 L/minute sweep gas flow. **C)** 19Fr catheter; 8 L/minute sweep gas flow. **D)** 19Fr catheter; 16 L/minute sweep gas flow. Fr, French.

**Figure 4:**
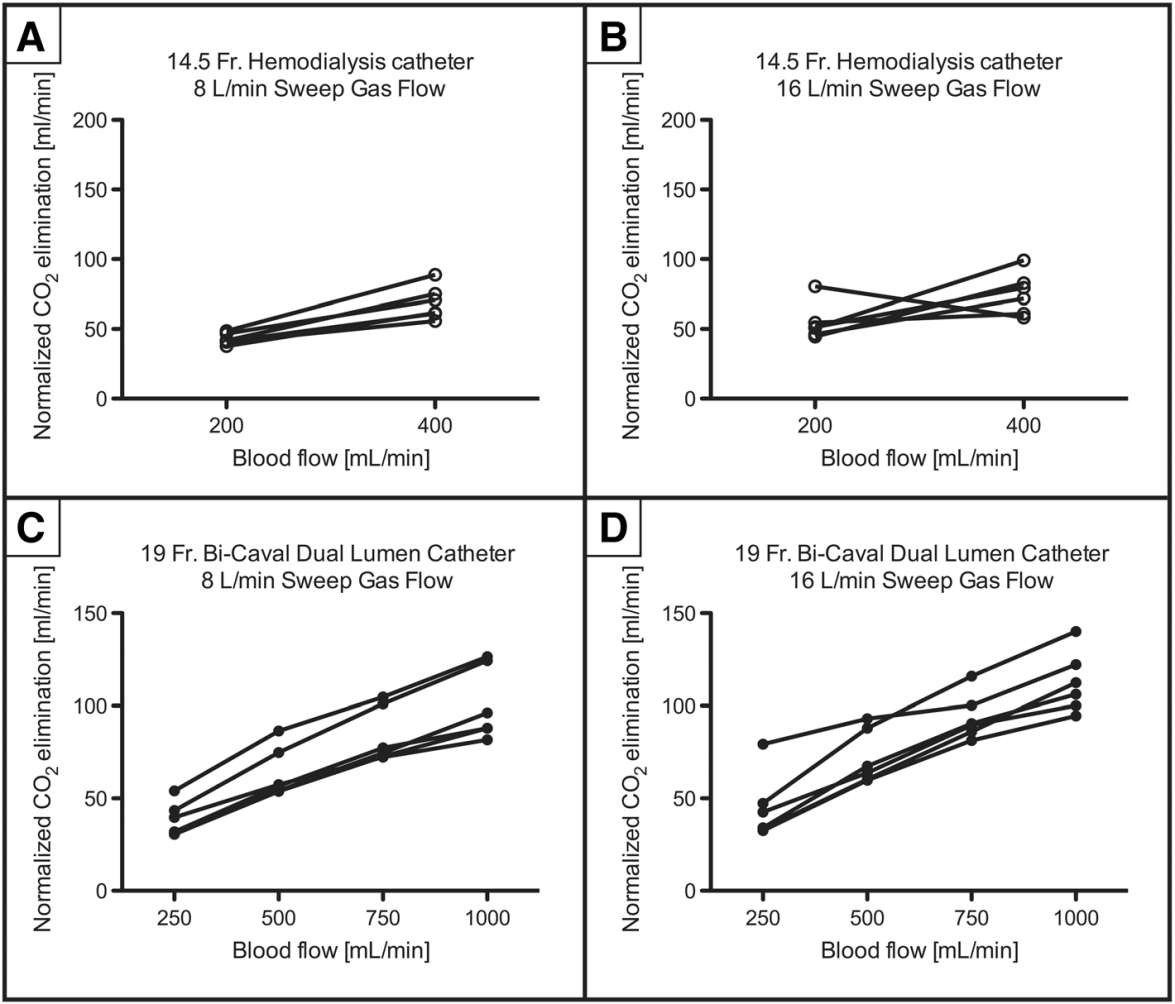
**Normalized elimination of carbon dioxide (CO**_**2**_**) depending on blood flow. A)** 14.5Fr catheter; 8 L/minute sweep gas flow. **B)** 14.5Fr catheter; 16 L/minute sweep gas flow. **C)** 19Fr catheter; 8 L/minute sweep gas flow. **D)** 19Fr catheter; 16 L/minute sweep gas flow.

ECCO_2_R was most effective with the 19Fr catheter at higher blood flow levels (experiments 3 and 4; Figure 1), with no clear difference between different sweep gas flow (8 versus 16 L/minute) conditions. In contrast, ECCO_2_R was less efficient with the 14.5Fr catheter (experiments 1 and 2; Figure 1). As a consequence, partial pressure of CO_2_ in arterial blood (PaCO_2_) progressively decreased with increasing blood flow when the 19Fr catheter, but not the 14.5Fr catheter, was used (Figure 2). Similarly, pH values progressively increased in proportion with blood flow with the 19Fr catheter, but not the 14.5Fr catheter (Figure 3). Of note, non-acidotic blood gas values were only achieved with 19Fr catheters at blood flow rates ranging from 750 to 1,000 ml/minute. Detailed data on CO_2_ elimination and related blood gas values relative to different blood flow levels at a sweep gas flow of 16 L/minute are provided in Tables 2 and 3 and Additional file 1, respectively. The absolute values of CO_2_ elimination were normalized to the partial pressure of CO_2_ in venous blood (PvCO_2_) and a partial pressure of CO_2_ (PCO_2_) value of 45 mmHg according to Wearden *et al*. [20] and are provided in Figure 4.

**Table 2:** 14.5Fr hemodialysis catheter, sweep gas flow 16 L/minute

**Blood flow [ml/min]**	**CO**_**2 **_**elimination [ml/min]**	**PaCO**_**2 **_**[mmHg]**	**pH**	**PaO**_**2 **_**[mmHg]**
**0**	0	91.3 ± 11.6	7.16 ± 0.07	137.1 ± 26.8
**200**	62.4 ± 12.2	85.6 ± 12.0	7.20 ± 0.06	167.6 ± 27.4
**400**	77.9 ± 17.4	82.3 ± 15.0	7.22 ± 0.07	173.3 ± 21.6

Fr, French; PaCO_2_, partial pressure of CO_2_ in arterial blood; PaO_2_, partial pressure of O_2_ in the arterial blood.

**Table 3:** 19Fr Bicaval Dual Lumen Catheter, sweep gas flow 16 L/minute

**Blood flow [ml/min]**	**CO**_**2 **_**elimination [ml/min]**	**PaCO**_**2 **_**[mmHg]**	**pH**	**PaO**_**2 **_**[mmHg]**
**0**	0	107.9 ± 20.7	7.13 ± 0.08	122.9 ± 29.4
**250**	83.4 ± 11.8	90.3 ± 13.5	7.20 ± 0.06	142.8 ± 35.8
**500**	118.7 ± 23.9	76.9 ± 9.6	7.27 ± 0.06	165.3 ± 39.8
**750**	138.3 ± 22.8	66.9 ± 11.1	7.34 ± 0.09	182.0 ± 42.6
**1,000**	146.1 ± 22.6	55.7 ± 10.5	7.41 ± 0.07	193.6 ± 41.0

**Figure 5:**
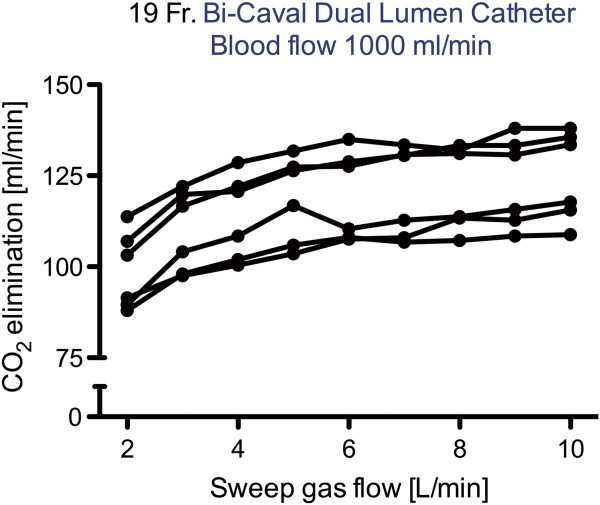
**Elimination of carbon dioxide (CO**_**2**_**) in dependence of sweep gas flow under a fixed flood flow of 1,000 ml/minute using a 19Fr catheter. Fr, French.**

**Figure 6:**
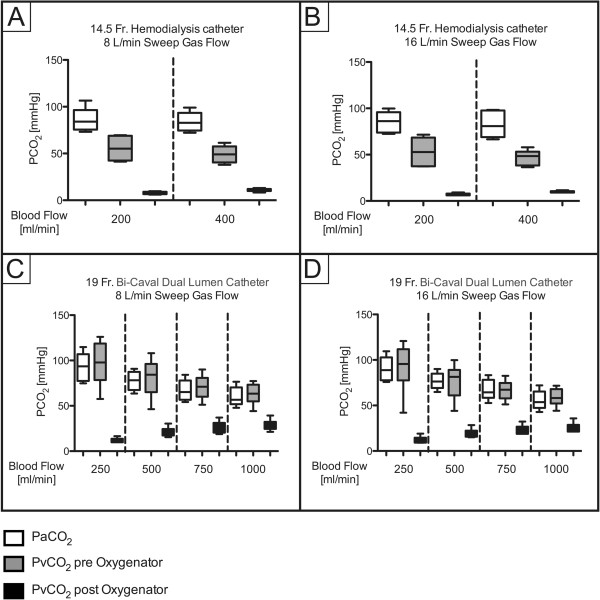
**Partial pressure of arterial (PaCO**_**2**_**) and venous (PvCO**_**2**_**) carbon dioxide depending on blood flow, sweep gas flow and cannula size. A)** 14.5Fr catheter; 8 L/minute sweep gas flow. **B)** 14.5Fr catheter; 16 L/minute sweep gas flow. **C)** 19Fr catheter; 8 L/minute sweep gas flow. **D)** 19Fr catheter; 16 L/minute sweep gas flow. Fr, French.

Corresponding data for different sweep gas flows were comparable, where a higher sweep gas flow of 16 L/minute resulted in only a slightly more effective ECCO_2_R compared to a lower sweep gas flow of 8 L/minute (Tables 2 and 3 and Additional file 1). However, a sweep gas flow below 6 L/minute resulted in less effective ECCO_2_R when large (19Fr) catheters were used at high blood flow levels (1,000 ml/minute) (Figure 5). The extracorporeal system was more effective with higher PvCO_2_ and lower blood flow levels and a longer oxygenator contact time, as demonstrated by a lower PCO_2_ post-oxygenator (Figure 6).

In three pigs, the sweep gas flow was switched from 100% oxygen to room air at the end of experiment 5, with blood flow rates of 1,000 ml/minute. This resulted in a further increase in CO_2_ removal (150 ± 26.6 versus 159.8 ± 22.6 ml/minute).

## Discussion

The present porcine study indicates that pump-driven veno-venous ECCO_2_R can normalize pH values and reduce PaCO_2_ in severe, life-threatening respiratory acidosis under constant ventilatory support. In addition, the present study suggests that a blood flow of 750 to 1,000 ml/minute is needed to achieve these results, since pH values remained acidotic at lower blood flow rates.

In particular, the severe respiratory acidosis in the model used in this study could not be sufficiently corrected either by ECCO_2_R at a blood flow of 200 to 400 ml/minute using the 14.5Fr catheter, or with blood flow rates of 500 ml/minute using the 19Fr cannula. In contrast, severe respiratory acidosis was normalized by veno-venous ECCO_2_R using the 19Fr catheter and a blood flow rate of 1,000 ml/minute, even though a blood flow of 750 ml/minute was sufficient in 50% of the animals. This finding contrasts with a recent study in human patients, where the application of a pump-driven veno-venous system using a 15.5Fr dual-lumen catheter was successful with a mean blood flow of 431 ml/minute [17]. Importantly, however, the mean pH in that study was around 7.25, thus considerably higher compared to the pH in the present study. Thus, based on the current findings, it remains questionable whether more acidotic pH values could also be successfully corrected by lower blood flow rates, although the 15.5Fr catheter has a more favorable design compared to the 14.5Fr Shaldon catheter. Therefore, using low-diameter catheters and low blood flow rates, pump-driven veno-venous ECCO_2_R may be primarily feasible in patients with mild to moderate respiratory acidosis. This may be aimed at reducing aggressiveness of invasive ventilation as originally, and also recently, described in patients with ARDS [7,9,12,21,22].

Interestingly, the ECCO_2_R capacity of the system used in the present study was in line with previous findings that were also derived from an animal study, where a removal-capacity up to 150 ml CO_2_/minute could be achieved with a blood flow of 1,000 ml/minute and an 18Fr catheter [23]. In accordance with the present trial, lower blood flow rates resulted in less efficient ECCO_2_R. In clear contrast, however, the mentioned trial provided evidence that ventilation parameters could be reduced following ECCO_2_R, but again, animals were not as severely acidotic as those in the present study. Therefore, the current study provides the first evidence that even severe acidosis can be successfully managed by ECCO_2_R and, in agreement with our clinical experience, this was only feasible with catheters that allowed blood flow rates of 750 to 1,000 ml/minute.

Since intensive care specialists are familiar with 14.5Fr hemodialysis catheters, it would be reasonable to test whether these catheters also qualify for ECCO_2_R. However, the maximal blood flow through these catheters is physiologically restricted to approximately 400 ml/minute. Furthermore, catheters specifically designed for ECCO_2_R aim to avoid recirculation [24,25]; this is particularly evident if the PCO_2_ of the venous blood, which is directed towards the oxygenator, is lower than arterial PCO_2_. Of note, recirculation was obvious when hemodialysis catheters were used, even with the lowest blood flow rates, demonstrated by a much lower CO_2_ before oxygenator than in the arterial blood (Figure 6A and B). In contrast, the specifically designed 19Fr catheter produced no significant recirculation, represented by higher CO_2_ before oxygenator than in arterial blood (Figure 6C and D). Therefore, (1) blood flow rates and (2) the specific technical design of the catheter that prevents recirculation are the main determinants of successful ECCO_2_R. Furthermore, CO_2_ elimination of the oxygenator depends closely on the diffusion gradient of CO_2_ between venous blood and the sweep gas. Therefore, with a higher CO_2_ content in venous blood, more CO_2_ will be eliminated, which has to be taken into consideration at the beginning of the experiments, when PvCO_2_ is highest. Furthermore, PaCO_2_ and PvCO_2_ were slightly higher in the low-flow group with the 19Fr than with the 14.5Fr catheter.

In the 1970’s, Kolobow [26] and Gattinoni [27] indicated that ECCO_2_R is dependent not only on venous PCO_2_, blood flow and sweep gas flow, but also on the size of the oxygenator. The currently used oxygenator provides a surface area of 0.98 m^2^ without heat exchange fibers. The high efficiency of the system is reflected by a very low mean PaCO_2_ value, leading to alkalotic pH values in the blood that is delivered by the oxygenator at low blood flow rates. This may explain why the previously described linear relationship between sweep gas flow and ECCO_2_R was not observed [22,24,28]. As shown by the present study, sweep gas flow rates of more than 6 L/minute, but not lower flow rates, can sufficiently maintain an optimal ECCO_2_R when a blood flow rate of 1,000 ml/minute is applied.

Interestingly, ECCO_2_R tended to be more efficient when 100% oxygen was switched to room air sweep gas. As hypercapnic respiratory failure is not necessarily associated with severe hypoxemia, the use of air as a sweep gas may slightly improve the effectiveness of ECCO_2_R, but will also reduce the oxygen transfer capacity of the extracorporeal support. On the other hand, the application of oxygen sweep gas was sufficient to improve oxygenation when the ratio of catheter blood flow rate to cardiac output was relatively high, in line with previous results [24].

The current study has some limitations. First, data acquired in animals cannot automatically be transferred into a clinical setting; however, the animals showed CO_2_ production and cardiac output rates that were similar to those observed in adult humans. Previous studies in humans have already shown the clinical effects of ECCO_2_R; thus, the present data are likely to be helpful in understanding the physiology of ECCO_2_R in humans as well. Furthermore, the anatomy of the pig is not comparable to a human adult, and the catheters we used may not be ideal in clinical practice. However, the perfect cannula for CO_2_ removal still has to be designed. Second, the typical clinical scenario of exacerbated COPD with severe airflow limitation was not simulated, since a rather high dead space ventilation protocol was used to artificially increase PaCO_2_. Therefore, the interaction between ECCO_2_R and mechanical ventilation could not be investigated. This, however, is suggested to be of clinical importance, since severe airflow limitation frequently requires the adaptation of ventilator settings. This, in turn, can lead to reduced alveolar ventilation that potentially impacts on acidosis. Third, the present study only applied short-term ECCO_2_R. In a real-life setting, the long-term effects of blood flow, cannula size and sweep gas flow application need to be elucidated, since CO_2_ is also stored in the form of HCO_3_ in slow compartments, and it may take several hours until a steady-state is reached. This is particularly pertinent to patients with acute-on-chronic ventilatory failure who present with respiratory acidosis, hypercapnia and high bicarbonate levels. On the other hand, the time constant for reducing blood CO_2_ in patients by ventilation is 10 minutes [29]; therefore, 15 minutes at each setting should have been an adequate amount of time to detect any changes in PaCO_2_. Furthermore, in the present study we did not put the focus on anticoagulation since clotting was no issue in our short-term experiments of 12 hours duration. Further studies have to be done in humans, since low-flow devices may need more anticoagulation in comparison to high-flow extracorporeal membrane oxygenation (ECMO). Finally, only one system for ECCO_2_R was tested in the present study, and this is suggested to limit the comparison of the current trial with other trials, in which different systems have been used. Nevertheless, the currently used system has shown to be highly effective for ECCO_2_R represented by a PCO_2_ lower than 10 mmHg and a PO_2_ of 600 mmHg after the oxygenator with low blood flows. Although other systems function in a different way, it is physically difficult to further lower PCO_2_. Thus, we believe that the present study is valid to systematically show changes in physiology during ECCO_2_R when using different conditions.

## Conclusions

In conclusion, this animal study has shown that severe respiratory acidosis can be successfully managed by veno-venous ECCO_2_R, but only when blood flow rates ranging between 750 and 1,000 ml/minute are applied. For this purpose, the use of specifically-designed catheters with an inner diameter greater than that of the typical hemodialysis catheters are required to establish flow rates ranging between 750 and 1,000 ml/minute and to avoid blood recirculation. In addition, the study has shown that a sweep gas flow rate above 6 L/minute is of minor importance for ECCO_2_R when using modern, highly-efficient oxygenators with low blood-flow rates. This holds true for the system tested, which is highly effective, represented by a PvCO_2_ of 10 to 30 mmHg after the oxygenator and, therefore, may be translated to comparable systems. This study also suggests that low-flow veno-venous ECCO_2_R with modern miniaturized membrane lungs can serve as a treatment option for severe respiratory acidosis associated with acute respiratory failure and severe hypercapnia. Whether these techniques prove to be suitable to avoid endotracheal intubation or to facilitate early extubation in intubated patients needs to be elucidated by future clinical trials.

## Key messages

• Severe respiratory acidosis with pH values of 7.0 to 7.2 can be successfully managed by veno-venous ECCO_2_R.

• For this purpose, blood flow rates ranging between 750 and 1,000 ml/minute are mandatory.

• Specifically-designed catheters aimed at avoiding blood recirculation warranting the target blood flow are required.

• Sweep gas flow rates above 6 L/minute are sufficient for maximal ECCO_2_R.

## Abbreviations

ARDS: acute respiratory distress syndrome; COPD: chronic obstructive pulmonary disease; ECCO_2_R: extracorporeal CO_2_ removal; ECMO: extracorporeal membrane oxygenation; Fr: French; PaCO_2_: partial pressure of CO_2_ in arterial blood; PALP: pump assisted lung protection; PCO_2_: partial pressure of CO_2_; PEEP: positive end expiratory pressure; PVCO_2_: partial pressure of CO_2_ in venous blood; NIV: non-invasive ventilation.

## Competing interests

CK received travel grants and lecture fees from Maquet, Rastatt, Germany. KK, GH and AL have no competing interests. FSS performs consultant services for Maquet Critical Care, Sweden. WW received fees for advisory board meetings and lectures from Maquet, Rastatt, Germany. TM received travel grants from Maquet, Rastatt, Germany. All authors declare that they have no non-financial competing interests.

## Authors’ contributions

CK designed the study (together with KA and TM). CK, FS and AL performed the animal experiments and analyzed the physiological data (together with TM and WW). GH designed and supervised the study and the analysis of results. WW and CK designed the concept of the manuscript; all the other authors (CK, KA, FS, AL, GH, TM) contributed to the final drafting of the manuscript. All authors read and approved the final manuscript.

## Supplementary Material

Additional file 1 **Absolute values of CO**_**2 **_**elimination and blood gas analysis with a sweep gas flow of 8 L O**_**2**_**/minute depending on blood flow.** The table shows the CO_2_ elimination capacity of the ECCO_2_R system and the corresponding blood gas analysis with a sweep gas flow of 8 L O_2_/minute with a hemodialysis catheter **(A)** and the 19Fr Bicaval Dual Lumen Catheter **(B)** according to different blood flow levels. It is clearly shown that ECCO_2_R is less efficient with the 14.5Fr catheter **(A)** compared to the 19Fr Bicaval Dual Lumen Catheter **(B)**. Click here for file
