# Supplementary material for: Veno-venous extracorporeal CO2 removal for the treatment of severe respiratory acidosis: pathophysiological and technical considerations
Source: Crit Care. 2014 Jun 17;18(3):R124. doi: 10.1186/cc13928 (PMC4095596; doi:10.1186/cc13928)
Supplement: Additional file 1 — Absolute values of CO2 elimination and blood gas analysis with a sweep gas flow of 8 L O2/minute depending on blood flow. The table shows the CO2 elimination capacity of the ECCO2R system and the corresponding blood gas analysis with a sweep gas flow of 8 L O2/minute with a hemodialysis catheter (A) and the 19Fr Bicaval Dual Lumen Catheter (B) according to different blood flow levels. It is clearly shown that ECCO2R is less efficient with the 14.5Fr catheter (A) compared to the 19Fr Bicaval Dual Lumen Catheter (B). [file cc13928-S1.doc]

**Additional file 1**

**A.** 14.5 Fr hemodialysis catheter, sweep gas flow 8 L/min

| **Blood flow [ml/min]** | **CO_2_ elimination [ml/min]** | **PaCO_2_ [mmHg]** | **pH** | **PaO_2_ [mmHg]** |
| --- | --- | --- | --- | --- |
| **0** | 0 | 91.3 ± 11.6 | 7.16 ± 0.07 | 137.1 ± 26.8 |
| **200** | 53.2 ± 14.9 | 86.2 ± 12.6 | 7.19 ± 0.06 | 156.4 ± 31.1 |
| **400** | 74.7 ± 17.0 | 83.9 ± 10.6 | 7.21 ± 0.05 | 173.5 ± 25.1 |

**B.** 19 Fr Bicaval Dual Lumen Catheter, sweep gas flow 8 L/min

| **Blood flow [ml/min]** | **CO_2_ elimination [ml/min]** | **PaCO_2_ [mmHg]** | **pH** | **PaO_2_ [mmHg]** |
| --- | --- | --- | --- | --- |
| **0** | 0 | 107.9 ± 20.7 | 7.13 ± 0.08 | 122.9 ± 29.4 |
| **250** | 79.2 ± 11.6 | 93.2 ± 16.4 | 7.19 ± 0.07 | 136.4 ± 34.4 |
| **500** | 111.3 ± 22.0 | 77.6 ± 11.2 | 7.26 ± 0.06 | 160.3 ± 38.0 |
| **750** | 129.4 ± 21.0 | 66.9 ± 11.7 | 7.32 ± 0.07 | 173.3 ± 38.3 |
| **1000** | 138.0 ± 17.0 | 59.9 ± 10.6 | 7.36 ± 0.06 | 188.7 ± 43.7 |
